# Malignant Potential of Thyroid Follicular Nodular Disease With Solid/Trabecular Components: A Case Report

**DOI:** 10.1111/pin.70058

**Published:** 2025-10-15

**Authors:** Mayu Ueda, Katsuya Matsuda, Yusuke Mori, Hirokazu Kurohama, Hiroyuki Mishima, Koh‐Ichiro Yoshiura, Norisato Mitsutake, Hisakazu Shindo, Shinya Sato, Hiroyuki Yamashita, Atsushi Kawakami, Masahiro Nakashima

**Affiliations:** ^1^ Department of Tumor and Diagnostic Pathology, Atomic Bomb Disease Institute Nagasaki University Nagasaki Nagasaki Japan; ^2^ Thyroid Cancer Collaborative Research Center, Atomic Bomb Disease Institute Nagasaki University Nagasaki Nagasaki Japan; ^3^ Department of Endocrinology and Metabolism Nagasaki University Graduate School of Biomedical Sciences Nagasaki Nagasaki Japan; ^4^ Yamashita Thyroid Hospital Fukuoka Fukuoka Japan; ^5^ Department of Human Genetics, Atomic Bomb Disease Institute Nagasaki University Nagasaki Nagasaki Japan; ^6^ Department of Molecular Oncology, Atomic Bomb Disease Institute Nagasaki University Nagasaki Nagasaki Japan

**Keywords:** Cancer gene panel, follicular thyroid carcinoma, nodule‐in‐nodule, ST components, *TERT* promoter, thyroid follicular nodular disease

## Abstract

Although rare, thyroid follicular nodular disease (TFND) may exhibit a nodule‐in‐nodule (NN) appearance with solid/trabecular (ST) components (STc). While the STc has histologically aggressive features compared to the outer nodule (Out‐N) in TFND, its pathological significance remains unclear. We present a case of TFND with STc in a 63‐year‐old man who developed skin implantation and lung metastases 3 years after lobectomy. Histologically, the skin tumor resembled STc with high mitotic activity. Molecular analysis revealed *EZH1* mutations in both the Out‐N and STc of TFND, while *KRAS* and *TERT* promoter mutations were restricted to STc and the skin tumor. These findings suggest that the STc of NN may be a precursor to poorly differentiated thyroid carcinoma arising from well‐differentiated components through stepwise mutations. This case highlights the malignant potential of certain Noninvasive TFNDs and suggests the need for further analyses to clarify this hypothesis and reconsider their classification and management.

## Graphical abstract

A case of noninvasive thyroid follicular nodular disease (TFND) with a solid/trabecular (ST) components (STc) developed skin implantation and lung metastases 3 years after lobectomy. The skin tumor resembled STc of the primary TFND and harbored *EZH1*, *KRAS* and *TERT* promoter mutations. This case highlights the malignant potential of STc in TFND.

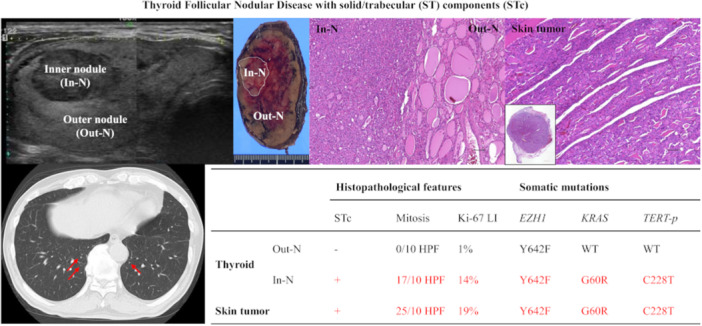

**Abbreviations**
53BP1: TP53‐binding protein 1
AG: adenomatous goiter
DDR: DNA damage response
FA: follicular adenoma
FNAC: fine‐needle aspiration cytology
In‐N: inner nodule
LI: labeling index
MAF: mutant allele frequency
mFTC: minimally invasive follicular thyroid carcinoma
NN: nodule‐in‐nodule
NTI: needle tract implantation
Out‐N: outer nodule
PDTC: poorly differentiated thyroid carcinoma
PTC: papillary thyroid carcinoma
STc: solid/trabecular components
TERT‐p: TERT promoter
TFND: thyroid follicular nodular disease
TFTs: thyroid follicular‐patterned tumors
WDTC: well‐differentiated thyroid carcinoma
wFTC: widely invasive follicular thyroid carcinoma

## 1 Introduction

The malignant potential of well‐differentiated thyroid follicular‐patterned tumors (TFTs) without papillary thyroid carcinoma (PTC)‐like nuclear features is typically evaluated based on capsular invasion, vascular invasion, and distant metastasis. The World Health Organization Classification 5th edition introduced the term thyroid follicular nodular disease (TFND) to accurately describe thyroid nodular lesions, including both hyperplastic and neoplastic nodules, which were previously diagnosed as adenomatous goiter (AG) and follicular adenoma (FA), respectively [1]. Recent evidence has also demonstrated that some of these nodules are clonal lesions, suggesting a potential risk of malignant transformation [2].

Although rare, TFTs can exhibit a nodule‐in‐nodule (NN) appearance with solid/trabecular (ST) components (STc), characterized by ST growth pattern, but lacking PTC‐like nuclear features, invasion, or metastasis. According to current diagnostic criteria, such nodules are generally classified as benign tumorous lesions, including TFND, despite histological similarity to poorly differentiated thyroid carcinoma (PDTC) in terms of increased cellularity, nuclear atypia, mitotic activity, and a high Ki‐67 labeling index (LI) [1]. Thus, although the STc exhibits histologically aggressive features compared to the outer nodule (Out‐N) in TFTs, its pathological significance remains unclear. PDTC is aggressive and reportedly evoked by TFTs. Our recent study demonstrated that the prevalence of *NRAS* codon 61 and *TERT* promoter (*TERT*‐p) mutations in STc is comparable to that in other carcinomas, including minimally invasive follicular thyroid carcinoma (mFTC) and widely invasive FTC (wFTC), suggesting that STc in NN is potentially a precursor lesion associated with PDTC [3].

Herein, we present a case of TFND with an NN appearance and STc that recurred and metastasized 3 years postoperatively, highlighting the clinical significance of identifying potentially malignant features in these nodules. To further characterize the molecular features of this tumor, we performed next‐generation sequencing analysis using our custom‐designed thyroid cancer gene panel.

## 2 Clinical Summary

**Figure 1:**
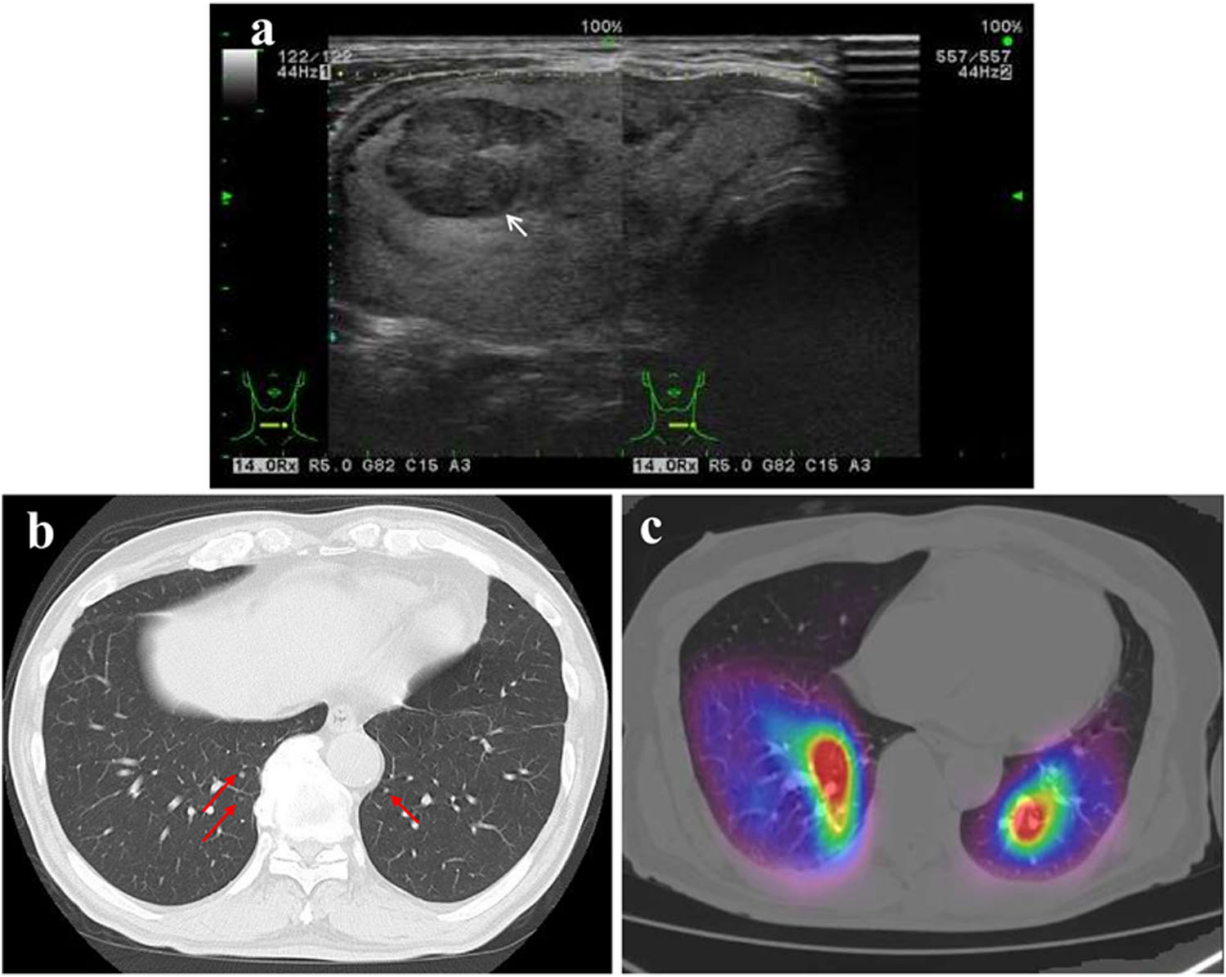
Clinical imaging findings. (a) Thyroid ultrasonography was performed before initial surgery, showing an isoechoic outer nodule and a hypoechoic inner nodule (white arrow). (b) Computed tomography performed after subcutaneous tumor enucleation suggests multiple small lung metastases. (c) 131‐I scan shows radioactive iodine uptake in the lungs.

A 63‐year‐old man was referred to our hospital with a thyroid nodule detected during physical examination. His medical history included hypertension and type 2 diabetes, with no family history of thyroid disease. Thyroid function tests were within normal limits (thyroid‐stimulating hormone: 0.66 μIU/mL; free T4: 1.09 ng/dL), but serum thyroglobulin was elevated (3854 ng/mL). Thyroid ultrasonography revealed an isoechoic mass measuring 80 mm in the right lobe, containing a well‐defined hypoechoic lesion measuring 26 mm (inner nodule) (Figure 1a). Fine‐needle aspiration cytology (FNAC) revealed a follicular neoplasm with oncocytic changes. Subsequently, right lobectomy was performed for nodule diagnosis and treatment, and no malignant findings were observed.

Three years postoperatively, the patient noticed a small subcutaneous nodule on the right side of the neck and underwent subcutaneous tumor enucleation. Computed tomography further revealed multiple small pulmonary nodules (Figure 1b), and serum thyroglobulin was significantly elevated (232 ng/mL) compared to the initial postoperative level (8.02 ng/mL). Complementary thyroidectomy was performed, and no neoplastic lesions were detected. Postoperatively, a whole‐body scan following the administration of radioactive 131‐I (3,700 MBq [100 mCi]) detected multiple pulmonary nodules (Figure 1c), suggesting lung metastasis from thyroid cancer.

## 3 Pathological Findings

**Figure 2:**
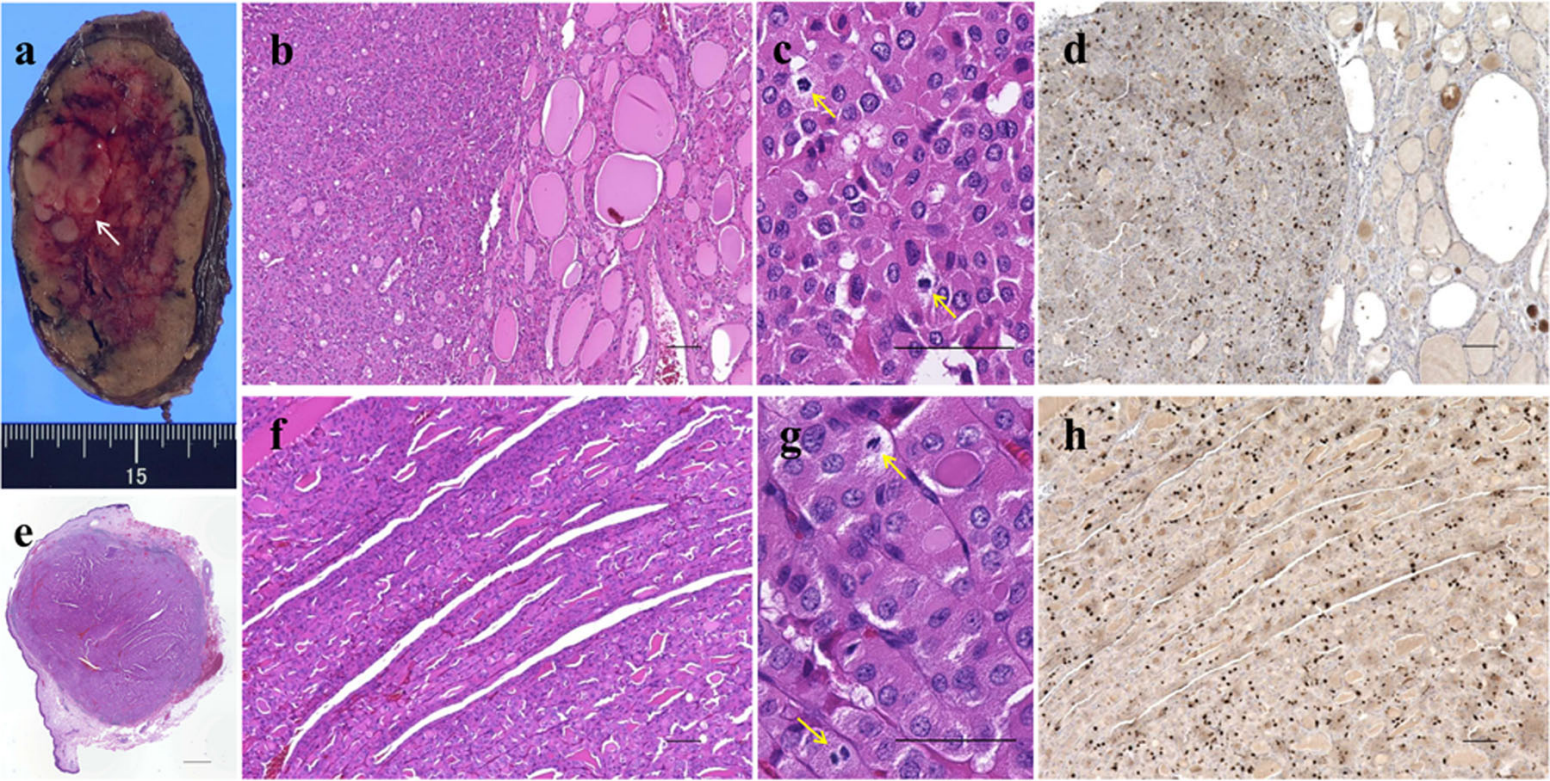
Histopathological appearances of the thyroid nodule and skin tumor. (a) Macroscopic image of the thyroid nodule. The outer thyroid nodule is well‐circumscribed, smooth‐margined, and partially hemorrhagic. The solid inner nodule (white arrow) is located within the outer nodule, with no evidence of invasion. (b–c) Histopathological features, and (d) proliferative activity determined by immunohistochemistry for Ki‐67 expression between the outer and inner nodules of the thyroid. The outer nodule shows a well‐differentiated follicular pattern and a few Ki‐67‐positive cells. In contrast, the inner nodule shows a microfollicular and solid growth pattern with oncocytic cytoplasm, some mitosis (yellow arrow), and several Ki‐67‐positive cells. (e–g) Loupe image, histopathological features, and (h) proliferative activity determined by immunohistochemistry for Ki‐67 expression of the skin tumor. A 7‐mm tumor in the dermis showing a small follicular and trabecular growth pattern with oncocytic cytoplasm, some mitosis (yellow arrow), and several Ki‐67‐positive cells. The scale bars are 50 μm (c, g), 100 µm (b, d, f, h), and 1 mm (e).

Histological examination of the thyroid nodule in the right lobe revealed a TFT with an NN appearance, lacking a capsule, invasive features, or necrosis. The Out‐N consisted of well‐formed follicles of various sizes showing oncocytic (eosinophilic) cytoplasm. The stroma exhibited edematous changes and hemorrhage. The inner nodule (In‐N) exhibited a microfollicular and solid growth pattern, oncocytic cytoplasm, and a round nucleus with a conspicuous nucleolus (Figure 2b). While mitotic features were absent in the Out‐N, the In‐N showed frequent mitotic features (17/10 high‐power field [HPF]) and a high Ki‐67 LI of 14% (Figure 2c,d). The lesion was diagnosed as an AG with an oncocytic STc, characterized by high mitotic features.

The subcutaneous tumor excised 3 years postoperatively was a 7‐mm dermal tumor with microfollicular and trabecular growth patterns, oncocytic cytoplasm, high mitotic activity (25/10 HPF), and a Ki‐67 LI of 19%, as well as a previously resected thyroid nodule, indicating a metastatic/implanted oncocytic FTC (Figure 2e–h).

## 4 Molecular Analyses

To characterize the molecular features of this tumor, next‐generation sequencing analysis was performed using a custom‐designed thyroid cancer gene panel. Genomic DNA was separately extracted from the Out‐N, In‐N, and the skin tumor using the Maxwell RSC DNA FFPE Kit (Promega, Madison, WI, USA) with the Maxwell RSC Instrument (Promega). Libraries were prepared from 50 ng of DNA using NEBNext FFPE DNA Repair Mix (Biolabs, Tokyo, Japan), NEBNext Ultra II DNA Library Prep Kit for Illumina (Biolabs), and xGen hybridization capture of DNA libraries (Integrated DNA Technologies, Tokyo, Japan), according to the manufacturer's protocol. The gene panel included the *TERT* promoter regions and all exon regions of the following genes: *AKAP9, AKT1, ALK, APC, ARID1A, ARID1B, ARID2, ATM, ATR, BCOR, BRAF, BRCA1, BRCA2, CDK4, CDKN1A, CDKN1B, CDKN2A, CHEK2, CTNNB1, DICER1, DNMT3A, EGFR, EIF1AX, EML4, ERBB2, ETV6, EZH1, FGFR1, GLIS3, GNAS, HRAS, IDH1, KIAA1549, KRAS, MED12, MLH1, MTOR, NF1, NF2, NRAS, NTRK1, NTRK3, PAX8, PARP1, PIK3CA, PPARG, PRKDC, PTEN, RAD51, RBM10, RET, RIF1, SQSTM1, STK11, STRN, TBL1XR1, TERT, TPR, TP53, TP53BP1, TPM3, TSHR, USP28*, and *XRCC4*. Based on DNA structural abnormalities, this panel could detect the following fusion genes: *ALK‐EML4, ALK‐STRN, BRAF‐AKAP9, BRAF‐KIAA1549, EGFR‐RAD51, NTRK1‐TPM3, NTRK1‐TPR, NTRK3‐ETV6, NTRK3‐SQSTM1, PAX8‐GLIS3, PAX8‐PPARG, PI3KCA‐TBL1XR1, RET‐CCDC6, RET‐NCOA4*, and *RET‐TBL1XR1*.

Paired‐end (150 bp × 2) sequencing was performed using HiSeqX Ten (Microgen, Tokyo, Japan), and somatic mutations with a ≥ 10% mutant allele frequency (MAF) were identified using Mutect2 and Strelka2. All variants were confirmed using IGV v2.18.4.

The *EZH1* Y642F mutation was detected in all tumor samples with variant allele frequency (VAF) 36.1%, 37.9%, and 18.9% in Out‐N, In‐N, and skin tumor, respectively. The *KRAS* G60R mutation was detected in In‐N (VAF 36.3%) and skin tumor (VAF 23.0%), while the *TERT*‐p *C228T* mutation was detected in In‐N (VAF 35.5%) and skin tumor (VAF 25.9%) **(**Table 1 **)**.

**Table 1:** Histopathological and genetic findings of the thyroid nodule and skin tumor.

		Histopathological features					Somatic mutations		
		Capsule	ST pattern	Cytoplasm	Mitosis	Ki‐67 LI	*EZH1*	*KRAS*	*TERT*‐p
Thyroid	Outer nodule	—	—	Oncocytic	0/10 HPF	1%	Y642F	WT	WT
	Inner nodule	—	+	Oncocytic	17/10 HPF	14%	Y642F	G60R	C228T
Skin tumor		—	+	Oncocytic	25/10 HPF	19%	Y642F	G60R	C228T

Abbreviations: HPF, high power field; LI, labeling index; ST, solid/trabecular; *TERT*‐p, *TERT* promoter; WT, wild type.

## 5 Discussion

Noninvasive TFTs exhibiting an NN appearance with STc are rare and have previously been described as indolent [4]. However, the present case indicates their malignant potential. In our previous study, we analyzed the expression profile of the DNA damage response (DDR) molecule TP53‐binding protein 1 (53BP1), *NRAS* codon 61, and *TERT*‐p mutations in 16 cases of TFTs with NN and STc, comparing them to 30 AGs, 31 FAs, 15 mFTCs, and 11 wFTCs. These results revealed that the expression level of abnormal type 53BP1, which is considered an indicator of altered DDR, and the incidence of *NRAS* and *TERT*‐p mutations in STc were comparable to those in FTCs, suggesting malignant potential [3]. Based on these findings, we hypothesized that STc development in NN may be associated with DDR impairment triggered by *NRAS* and *TERT*‐p mutations.

Compared with our previous 16 cases of NN, the present case was characterized by a larger tumor size and higher proliferative activity. In our earlier series, the mean sizes of Out‐N and In‐N were 42 and 13 mm, respectively, with an average mitotic count of 3.6/10 HPF. In contrast, the current case showed an Out‐N of 80 mm, an In‐N of 26 mm, and 17 mitoses/10 HPF, all of which were markedly higher. Furthermore, the frequencies of the 53BP1 abnormal type (33.4%) and the 53BP1 and Ki‐67 double‐positive type (2.4%) in In‐N of this case (Supplemental Figure 1) were substantially higher than those in our previous report (10.3 ± 5.5% and 0.36 ± 0.40%, respectively), representing the highest levels among the 16 reported cases. These findings suggest enhanced genomic instability and support our hypothesis. In addition to this case, one previous case among our 16 cases showed oncocytic changes, but it did not harbor TERT promoter C228T/C250T mutations, was small in size (Out‐N 31 mm/In‐N 7 mm), had low mitotic activity (0/10 HPF), and also showed low 53BP1 expression (53BP1 abnormal type 8.1%/53BP1+Ki‐67 double‐positive type 0.74%).

In the current case, histopathological differences between the Out‐N and In‐N (oncocytic STc) were evident, particularly in terms of ST pattern, mitotic count, and Ki‐67 LI. The skin tumor was histologically identical to In‐N and exhibited mutations, including *EZH1* Y642F, *KRAS* G60R, and *TERT*‐p *C228T*, which were also identified in In‐N. Therefore, we concluded that the skin tumor was metastatic or implanted from the In‐N. Metastatic skin tumor from well‐differentiated thyroid carcinoma (WDTC), which typically occurs on the scalp, is extremely rare [5]. In the present case, a skin tumor was found on the right side of the neck, which could have been a local recurrence due to needle tract implantation (NTI) during FNAC [6]. NTI is more common in tumors with aggressive features [7]. Although no biopsy was performed on the lung nodules, accumulation of 131‐I was observed, suggesting metastatic thyroid carcinoma. A previous report also described a case of noninvasive FA with solid/trabecular/insular pattern, necrosis, and high mitotic features with multiple metastases [8].

Molecular analysis detected three mutations in *EZH1*, *KRAS*, and *TERT*‐p in In‐N and one in *EZH1* in the Out‐N. These findings support our hypothesis that STc in NN may arise from Out‐N via the acquisition of *RAS* and *TERT*‐p mutations. In thyroid tumors, *EZH1* seems to be associated with oncocytic tumorigenesis [9], with frequencies of 20% and 10% in oncocytic adenomas and carcinomas, respectively [9]. *EZH1* Y642F mutation has also been identified in a subpopulation of these cases [9]. Both Out‐N and In‐N showed oncocytic histology, suggesting that *EZH1* Y642F may act as a driver, with *RAS* and *TERT*‐p mutations promoting malignant transformation. *EZH1* is a component of polycomb repressive complex 2, which is involved in the dysregulation of transcriptional repression by methylating histone H3 at Lys27 [10]. Immunohistochemical analysis of H3K27me3 in this case revealed slightly lower expression in In‐N than in stromal cells, used as an internal control (Supplemental Figure 2). Previous studies have reported that *EZH1* Q571R mutations are associated with increased H3K27me3 levels [10], and that *EZH1* mutation–induced H3K27me3 overexpression correlates with tumor aggressiveness and dedifferentiation in thyroid carcinoma [11]. However, the effect of the *EZH1* Y642F mutation on H3K27me3 expression remains unclear. In other cancers, such as breast carcinoma, loss of H3K27me3 has been linked to poor prognosis [12]. Therefore, the relationship between *EZH1* mutations, H3K27me3 status, and their oncological significance warrants further investigation. Notably, the *KRAS* G60R mutation has not previously been reported in thyroid tumorigenesis. Further studies are needed to clarify its role in this rare tumor type. *TERT*‐p mutations are frequently detected in PDTC but are rare in benign thyroid nodules and WDTC [13, 14, 15, 16]. A previous report described a case of FA with a *TERT*‐p mutation that recurred 3 years after initial surgery, suggesting that *TERT*‐p mutations may represent an early genetic event preceding the development of histopathological malignant features in TFTs [16]. Our case further reinforces the significance of *TERT*‐p mutation in the poor differentiation/presence of STc and metastatic capability during thyroid tumorigenesis.

In conclusion, this report describes a rare case of TFND presenting as a noninvasive TFT with an NN appearance and STc, which recurred in the skin and lung 3 years postoperatively. Molecular profiling using our custom thyroid cancer gene panel revealed the molecular characteristics of this tumor, which showed additional mutations (*KRAS* and *TERT*‐p) in In‐N, indicating STc with malignant potential from the well‐differentiated Out‐N. STc in the NN may be a precursor lesion associated with PDTC. Further analyses are required to clarify this hypothesis and establish a new classification of TFND.

## Author Contributions

M.U., H.K., and M.N. confirmed the pathological diagnoses; Y.M., H.S., S.S., and H.Y. treated the patients; M.U., K.M., H.M., K.Y., and N.M. performed genetic analysis; and M.U., K.M., and M.N. wrote and revised the manuscript. A.K. supervised the study. All the authors have read and agreed to the published version of the manuscript.

## Ethics Statement

This study was conducted in accordance with the tenets of the Declaration of Helsinki and approved by the Institutional Ethical Committee for Medical Research at Nagasaki University (approval date: April 28, 2023; #15062617‐6).

## Conflicts of Interest

The authors declare no conflicts of interest.

## Supporting information


**Figure 1:** Dual‐color immunofluorescence for TP53‐binding protein 1 (53BP1, green) and Ki‐67 (red) in the In‐N. **Figure 2:** Immunohistochemistry for H3K27me3.

## Data Availability

The data that support the findings of this study are available from the corresponding author upon reasonable request.
