# Supplementary material for: Malignant Potential of Thyroid Follicular Nodular Disease With Solid/Trabecular Components: A Case Report
Source: Pathol Int. 2025 Oct 15;75(12):624–8. doi: 10.1111/pin.70058 (PMC12747683; doi:10.1111/pin.70058)
Supplement: Supplementary file 1 — Figure 1: Dual‐color immunofluorescence for TP53‐binding protein 1 (53BP1, green) and Ki‐67 (red) in the In‐N. Figure 2: Immunohistochemistry for H3K27me3. [file PIN-75-624-s001.pptx]

## Slide 1
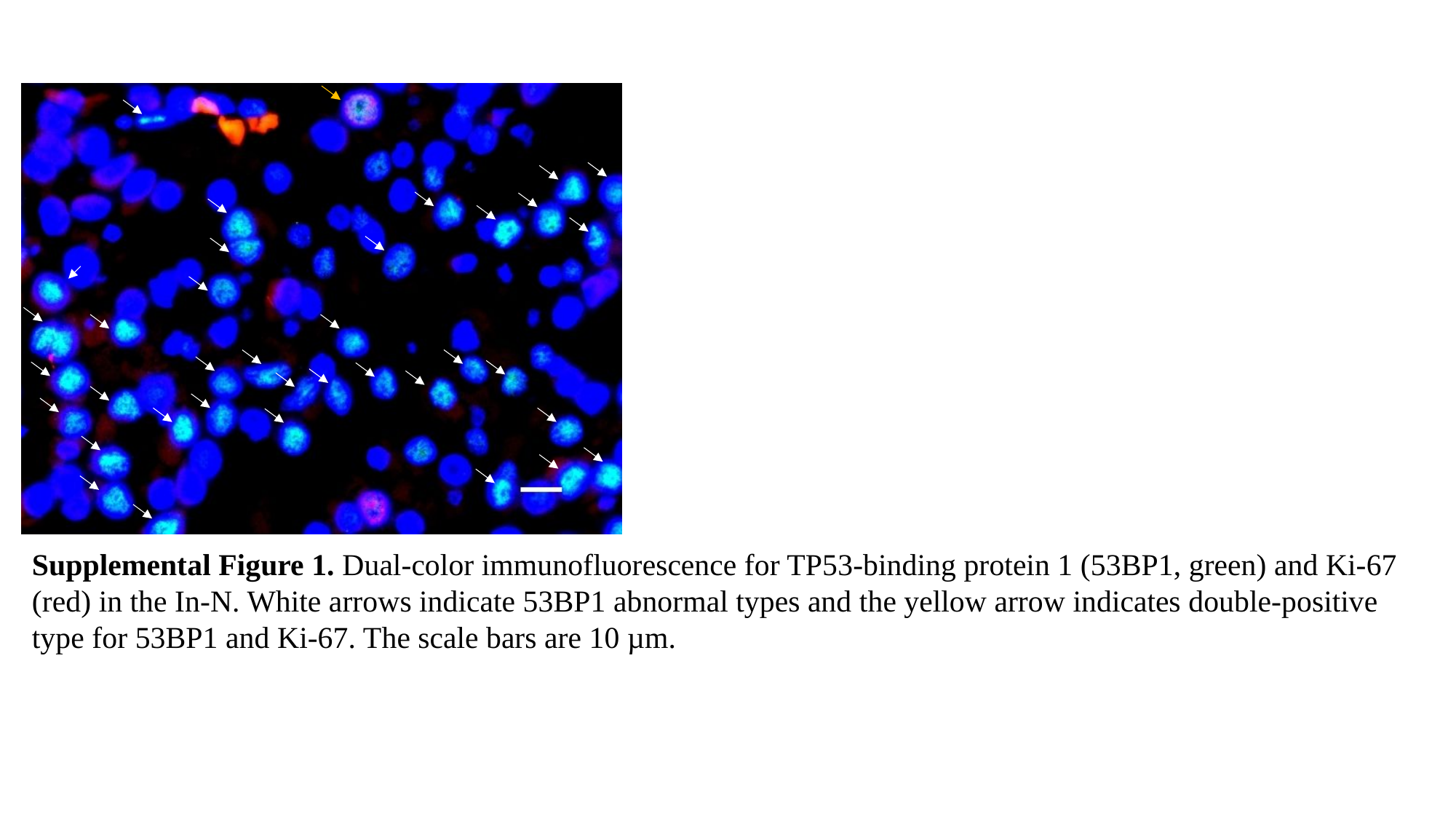

Supplemental Figure 1. Dual-color immunofluorescence for TP53-binding protein 1 (53BP1, green) and Ki-67 (red) in the In-N. White arrows indicate 53BP1 abnormal types and the yellow arrow indicates double-positive type for 53BP1 and Ki-67. The scale bars are 10 µm.

## Slide 2
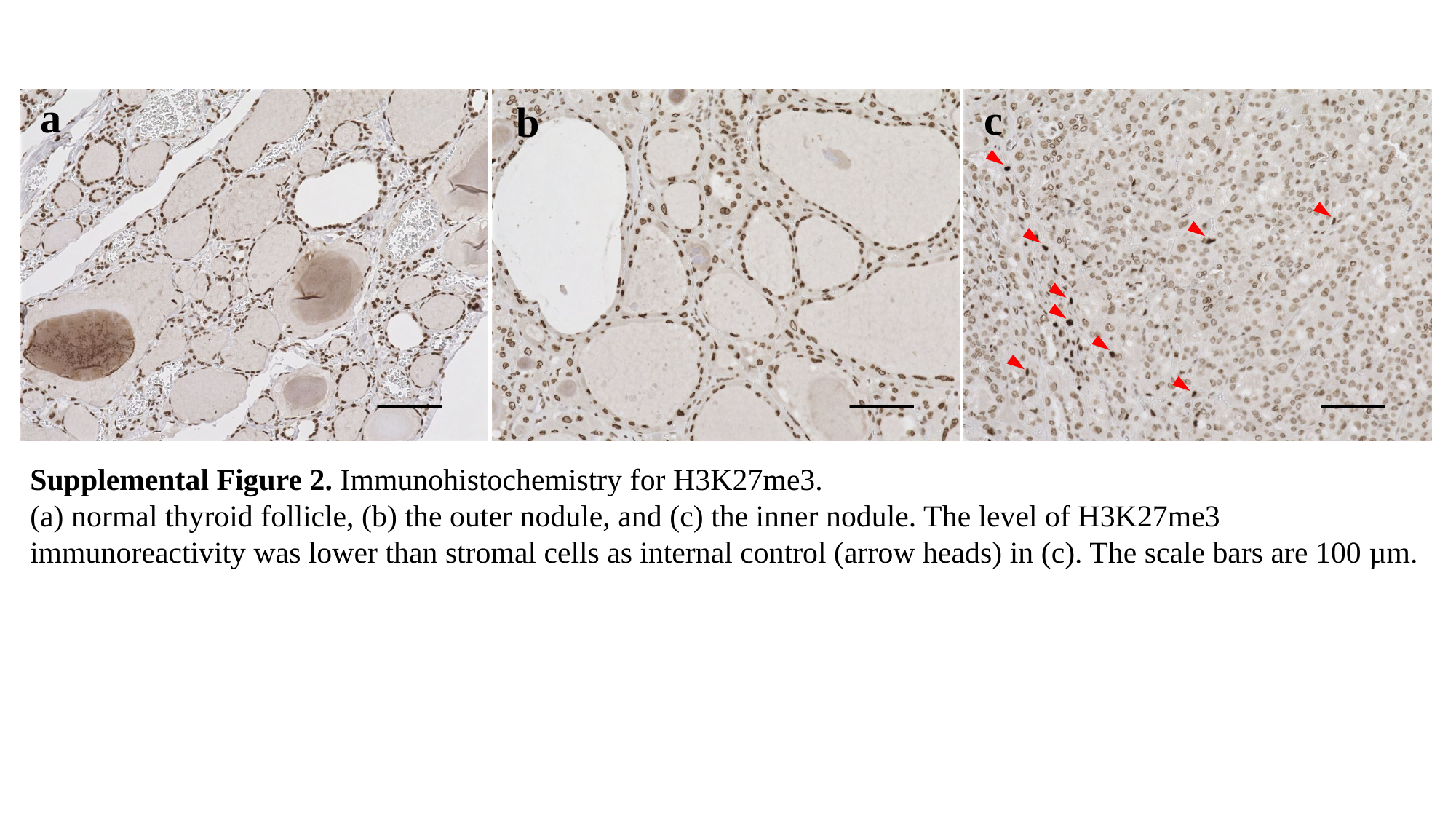

a
c
b
Supplemental Figure 2. Immunohistochemistry for H3K27me3.
(a) normal thyroid follicle, (b) the outer nodule, and (c) the inner nodule. The level of H3K27me3 immunoreactivity was lower than stromal cells as internal control (arrow heads) in (c). The scale bars are 100 µm.
